# Risk factors for hospitalization in pediatric patients with human adenovirus infection and changes in epidemiological and clinical characteristics before and after the COVID-19 pandemic in China: a multicenter retrospective study

**DOI:** 10.3389/fcimb.2026.1863975

**Published:** 2026-08-05

**Authors:** Xuan Xu, Min Kong, Xiaojun Zhang, Jiangning Yin, Xiu Chen, Li Xie, Fangling Wei, Jianguo Zhang, Zhimin Tao

**Affiliations:** 1 Department of Emergency Medicine, The Affiliated Hospital, Jiangsu University, Zhenjiang, Jiangsu, China; 2 Department of Emergency Medicine, The First People’s Hospital of Kunshan, Affiliated Kunshan Hospital of Jiangsu University, Kunshan, Jiangsu, China; 3 Department of Emergency Medicine, The People’s Hospital of Danyang, Affiliated Danyang Hospital of Nantong University, Danyang, Jiangsu, China; 4 Department of Emergency Medicine, The Affiliated Jiangning Hospital of Nanjing Medical University, Nanjing, Jiangsu, China; 5 Department of Pediatrics, The People’s Hospital of Danyang, Affiliated Danyang Hospital of Nantong University, Danyang, Jiangsu, China; 6 Jiangsu Province Key Laboratory of Medical Science and Laboratory Medicine, Department of Laboratory Medicine, School of Clinical Medicine and Laboratory Medicine, Jiangsu University, Zhenjiang, Jiangsu, China

**Keywords:** COVID-19 pandemic, epidemiology, human adenovirus, pediatric patients, risk factors

## Abstract

**Background:**

Human adenovirus (HAdV) is an important cause of respiratory and systemic infection in children. Since the COVID-19 pandemic, changes in population immunity, transmission patterns, and circulation of respiratory pathogens may have altered the epidemiological and clinical profile of pediatric HAdV infection. However, data comparing pediatric HAdV infection before and after the COVID-19 pandemic remain limited.

**Methods:**

We analyzed pediatric patients with HAdV infection admitted to four tertiary hospitals in Jiangsu Province, China, before and after the COVID-19 pandemic. Epidemiological features, including seasonal distribution and age patterns, were compared between the two periods. Clinical manifestations, co-infections, inflammatory biomarkers, and imaging findings were evaluated to characterize differences in disease presentation between pre- and post-COVID periods. Also, risk factors associated with pediatric hospitalization in the post-COVID period were explored.

**Results:**

Marked changes were observed in the epidemiological and clinical profile of pediatric HAdV infection after the COVID-19 pandemic. In the post-COVID period, hospitalized children were younger than outpatients and were more likely to present with respiratory, gastrointestinal, and neurological symptoms. They had higher rates of viral or bacterial co-infections, particularly influenza co-infection, along with moderately increased inflammatory biomarkers and greater pulmonary involvement. In addition, seasonal patterns and age distribution shifted measurably after the COVID-19 pandemic. Despite these changes, the overall clinical severity of pediatric HAdV infection did not show substantial worsening.

**Conclusion:**

Pediatric HAdV infection after the COVID-19 pandemic was characterized by a distinct epidemiological and clinical pattern, including altered seasonality, younger age concentration, increased influenza co-infection, and moderately heightened inflammatory responses. However, these changes were not accompanied by a substantial deterioration in overall clinical severity. Continued surveillance is warranted to monitor the evolving post-pandemic profile of pediatric HAdV infection.

## Introduction

Circulating worldwide, human adenoviruses (HAdVs) are non-enveloped double-stranded DNA viruses in the *Adenoviridae* family that may infect the respiratory, gastrointestinal, ocular, and urinary tracts (Shieh, 2022). Due to genetic diversification through mutation, recombination, and evolutionary adaptation, HAdVs exhibit multiple genotypes that vary in antigenicity, tissue tropism and disease severity (MacNeil et al., 2023). As a result, more than 100 human adenovirus genotypes have been identified and divided into seven species (A through G), each owning distinct clinical manifestations and epidemiological patterns (Kajon, 2024). For instance, HAdV-41 variants (F species) were suspected to be the causative pathogen for acute hepatitis in children (Sanchez et al., 2022; Martin et al., 2023). HAdV-7 (B species) has been frequently linked to severe respiratory infections with a basic reproductive number of 5.1 (Guo et al., 2020), causing community outbreaks with pandemic potential (Radke and Cook, 2023).

Although HAdVs can infect individuals of all ages and most infections are self-limiting in healthy individuals, this virus contributes substantially to the burden of acute respiratory infections in the pediatric population who may develop severe respiratory illness (e.g., pneumonia), sometime progressing to life-threatening sepsis and even death (Xie et al., 2019; Zhao et al., 2022; Xu et al., 2023). Several factors may contribute to the heightened vulnerability of children, including longtime aggregate in closed environment (i.e., daycare/school clustering), immaturity of immune system, and lack of pre-existing neutralizing antibodies (Lynch and Kajon, 2016). Thus, HAdV infections represent a substantial cause of hospitalization and morbidity among pediatric patients, where disease severity is associated with a variety of risk factors, including heightened viral loads, elevated white blood cell (WBC) count, and increased serum levels of C-reactive protein (CRP), procalcitonin (PCT) and lactate dehydrogenase (LDH) (Esposito et al., 2016; Shen et al., 2019; Goikhman et al., 2020; Huang et al., 2025).

Previous studies on pediatric infections of HAdVs have largely been limited to single-center investigations with relatively small sample sizes and were predominantly conducted prior to the COVID-19 pandemic (Esposito et al., 2016; Shen et al., 2019; Wu et al., 2020; Xu et al., 2023). Widespread implementation of non-pharmaceutical interventions (NPIs) during the COVID-19 pandemic, including mask mandates, travel restrictions, and social distancing, substantially altered the epidemiological landscape of many respiratory viruses (Ye et al., 2024; Zhang et al., 2024; Zhang Y. et al., 2025). How the COVID-19 pandemic has reshaped the epidemiology of pediatric adenovirus infections remains understudied, which would be otherwise important for predicting future epidemic patterns and improving public health preparedness. In parallel, studies examining risk factors for hospitalization among pediatric patients with HAdV infection in the post-COVID era are scarce (Soler Wenglein et al., 2025b).

Considering these knowledge gaps, this multicenter retrospective study included 3,045 pediatric patients with laboratory-confirmed HAdV infection from four tertiary hospitals in Jiangsu Province, China. Cases were collected from January 2015 to December 2019 (pre-COVID period) and from November 2023 to December 2025 (post-COVID period), with their clinical data analyzed, including baseline characteristics, laboratory results, radiological findings, and medication histories. We first compared outpatients and inpatients to identify risk factors associated with hospitalization in the post-COVID period, followed by comparison of hospitalized pediatric patients between the pre- and post-COVID periods to evaluate alterations in their epidemiological and clinical characteristics. This study aims to clarify whether pediatric adenovirus infection patterns have changed after the unprecedented COVID-19 pandemic and to provide evidence supporting the improved clinical management and public health preparedness.

## Methods

### Study design

**Figure 1:**
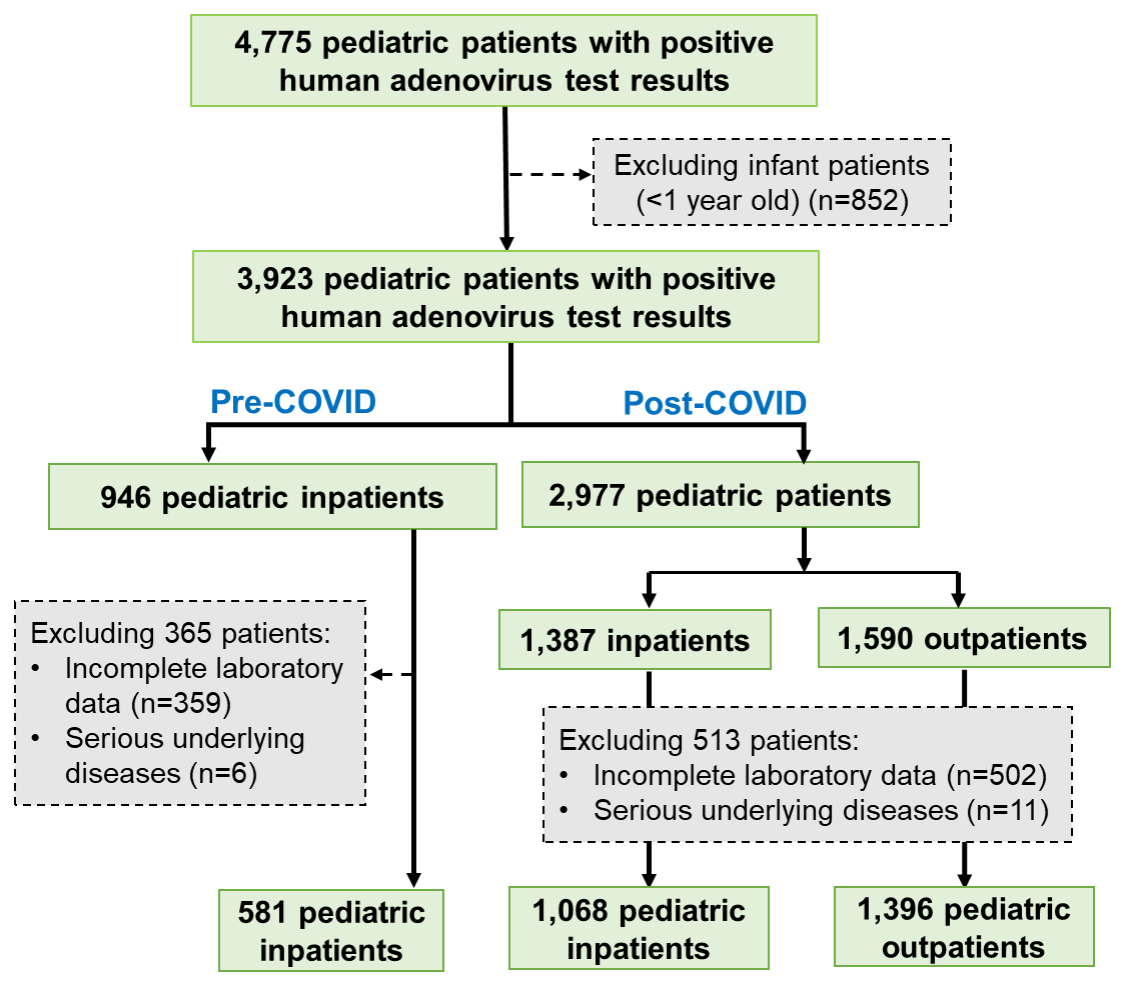
Inclusion and exclusion criteria of pediatric patients infected with HAdV.

This multicenter retrospective observational study included pediatric patients with confirmed HAdV infection from four tertiary and university-affiliated teaching hospitals in Jiangsu Province, China, including the Affiliated Hospital of Jiangsu University (AHJU) in Zhenjiang, the Affiliated Jiangning Hospital to Nanjing Medical University (AJHNMU) in Nanjing, the First People’s Hospital of Kunshan (FPHK) in Suzhou, and the People’s Hospital of Danyang (PHD). The study period spanned from January 1, 2015, to December 31, 2019 (pre-COVID group) and from November 1, 2023, to December 31, 2025 (post-COVID group). The inclusion and exclusion procedures of pediatric patients were illustrated in **Figure 1**.

For patients with repeated HAdV-positive results during the same illness episode, only the first positive test was retained for analysis. When both outpatient and inpatient encounters occurred within the same episode, the case was classified according to the highest level of care received, and duplicate records were removed. A new infection episode was defined only when a subsequent positive HAdV test occurred after documented clinical recovery or a symptom-free period of at least 30 days. Exclusion criteria included patients with age <1 year (due to that their hematological reference ranges differ significantly from those in children aged 1-18 years), severe underlying conditions (e.g., hematologic malignancies, congenital immunodeficiency, or others) that may influence the blood panel data, and incomplete clinical records.

The post-COVID group comprised 1,396 outpatients and 1,068 inpatients, where their clinical characteristics were analyzed and compared to derive the risk factors associated with hospitalization. Then 1,068 inpatients in the post-COVID group were compared to 581 hospitalized patients in the pre-COVID group to derive differences in the epidemiological and clinical profiles of pediatric inpatients with HAdV infection since the COVID-19 pandemic. Laboratory tests were performed at the four participating hospitals according to their routine diagnostic procedures, using institution-specific platforms, commercial reagents, and quality-control systems. Positive results were determined according to manufacturers’ instructions and institutional laboratory standards, and data were extracted directly from laboratory reports without reinterpretation.

The study was conducted in accordance with the Declaration of Helsinki and approved by the Research Ethics Committees of all participating hospitals. The requirement for informed consent was waived due to the retrospective nature of this study.

### Diagnosis of HAdV infection

The diagnosis of HAdV infection was following the previous publication (Wang N. et al., 2024). In our study, HAdV infection was confirmed using antigen-based or nucleic acid-based diagnostic methods. Immunofluorescence assay and colloidal gold assay detect HAdV viral antigens, whereas real-time PCR detects HAdV viral DNA. The distribution of diagnostic methods differed significantly between the pre- and post-COVID periods: immunofluorescence assay was used in 336 patients (57.8%) before COVID-19 and 1,848 patients (75.0%) after COVID-19; colloidal gold assay was used in 245 patients (42.2%) before COVID-19 and 369 patients (15.0%) after COVID-19; and real-time PCR was used only in the post-COVID period, in 247 patients (10.0%) (all *p* < 0.001).

HAdV and other respiratory pathogens were generally tested concurrently using routine multiplex panels. Depending on the hospital, an eight- or nine-pathogen panel was used. Co-infection analyses were limited to pathogens consistently included across panels, mainly influenza A/B viruses and *Mycoplasma pneumoniae*. Other pathogens with detection rates below 5% were not analyzed. Testing was performed as part of routine clinical care at the discretion of attending physicians rather than according to a standardized screening protocol.

### Statistical analysis

All statistical analyses were performed using SPSS version 27.0. Continuous variables are presented as medians with interquartile ranges (IQRs), and categorical variables as frequencies. Differences between the two groups were assessed using the Mann-Whitney U test for continuous variables and the χ^2^ test for categorical variables. Binary logistic regression analysis was used to evaluate factors associated with the outcome of interest. All statistical tests were two-sided, and *p* < 0.05 was considered statistically significant.

## Results

### Clinical characteristics of pediatric patients with HAdV infection in the post-COVID period

The post-COVID group included 2,464 pediatric patients with HAdV infection, where 1,396 were outpatients (56.7%) and 1,068 were inpatients (43.3%) (**Table 1**). Their median age was 5 years (IQR: 4-7) with 47.8% females. Inpatients were younger than outpatients, and toddlers (children aged 1-3 years) were more frequently represented among those hospitalized. Adolescents (≥14 years) accounted for only a small proportion of the study population, whereas children aged >3 to <14 years comprised the majority. In addition, gender ratio was similar between two patient groups.

**Table 1:** Baseline demographic, clinical characteristics and laboratory parameters of outpatient and inpatient children with HAdV infection in the post-COVID period.

Characteristic	Total (n=2,464)	Outpatient (n=1,396)	Inpatient (n=1,068)	p
Age (year)	5 (4-7)	6 (4-8)	5 (3-7)	<0.001
1≤Age ≤ 3, N (%)	549 (22.3%)	239 (17.1%)	310 (29.0%)	<0.001
3<Age<14, N (%)	1897 (77.0%)	1146 (82.1%)	751 (70.3%)	<0.001
Age≥14, N (%)	18 (0.7%)	11 (0.8%)	7 (0.7%)	0.702
Female sex, N (%)	1179 (47.8%)	674 (48.3%)	505 (47.3%)	0.624
Symptoms				
Fever	2282 (92.6%)	1337 (95.8%)	945 (88.5%)	<0.001
Cough	1352 (54.9%)	615 (44.1%)	737 (69.0%)	<0.001
Sputum	677 (27.5%)	126 (9.0%)	551 (51.6%)	<0.001
Sniffle	464 (18.8%)	243 (17.4%)	221 (20.7%)	0.039
Vomiting	352 (14.3%)	144 (10.3%)	208 (19.5%)	<0.001
Sore throat	309 (12.5%)	186 (13.3%)	123 (11.5%)	0.180
Abdominal pain	184 (7.5%)	87 (6.2%)	97 (9.1%)	0.008
Diarrhea	94 (3.8%)	51 (3.6%)	43 (4.0%)	0.632
Muscle pain	75 (3.0%)	62 (4.4%)	13 (1.2%)	<0.001
Convulsion	44 (1.8%)	5 (0.4%)	39 (3.7%)	<0.001
Rash	33 (1.3%)	8 (0.6%)	25 (2.3%)	<0.001
Co-infection				
Mycoplasma pneumoniae	342 (13.9%)	57 (4.1%)	285 (26.7%)	<0.001
Influenza B virus	234 (9.5%)	9 (0.6%)	225 (21.1%)	<0.001
Influenza A virus	187 (7.6%)	31 (2.2%)	156 (14.6%)	<0.001
Respiratory syncytial virus	47 (1.9%)	15 (1.1%)	32 (3.0%)	<0.001
Chlamydia	33 (1.3%)	14 (1.0%)	19 (1.8%)	0.097
Number of co-infections				
0	1879 (76.3%)	1278 (91.6%)	601 (56.3%)	<0.001
1	382 (15.5%)	110 (7.9%)	272 (25.5%)	<0.001
2	131 (5.3%)	8 (0.6%)	123 (11.5%)	<0.001
3+	72 (3.0%)	0	72 (6.7%)	<0.001
Underlying diseases				
Asthma	35 (1.4%)	1 (0.1%)	34 (3.2%)	<0.001
Allergic rhinitis	26 (1.1%)	0	26 (2.4%)	<0.001
Congenital heart defects	4 (0.2%)	0	4 (0.4%)	0.074
Peripheral blood parameters				
WBCs, ×10^9^/L	10.0 (7.2-12.9)	10.6 (8.3-13.2)	8.9 (6.0-12.2)	<0.001
Neutrophils, ×10^9^/L	6.7 (4.3-9.5)	7.5 (5.4-10.0)	5.2 (3.0-8.4)	<0.001
Lymphocytes, ×10^9^/L	2.0 (1.5-2.7)	1.9 (1.5-2.4)	2.2 (1.6-3.1)	<0.001
Monocytes, ×10^9^/L	0.8 (0.6-1.1)	0.9 (0.7-1.2)	0.6 (0.4-1.0)	<0.001
Red blood cells, ×10^12^/L	4.5 (4.3-4.8)	4.6 (4.4-4.9)	4.4 (4.2-4.6)	<0.001
Hemoglobin, g/L	124 (118-131)	127 (121-133)	120 (114-126)	<0.001
HCT, %	38.9 (36.5-41.2)	40.5 (38.8-42.3)	36.6 (34.7-38.4)	<0.001
MCV, fL	85.7 (82.8-88.5)	87.5 (85.2-89.8)	83.3 (81.3-85.6)	<0.001
MCH, pg	27.4 (26.6-28.2)	27.5 (26.7-28.3)	27.3 (26.5-28.1)	<0.001
MCHC, g/L	318 (312-326)	314 (309-320)	326 (319-334)	<0.001
RDW, %	12.9 (12.5-13.4)	13.0 (12.6-13.5)	12.8 (12.3-13.3)	<0.001
Platelets, 10^9^/L	254 (212-305)	249 (212-296)	259 (213-319)	<0.001
PDW, %	12.6 (10.4-15.9)	13.2 (10.4-15.9)	12.0 (10.3-15.8)	<0.001
CRP, mg/L	12.7 (4.5-26.4)	12.3 (4.5-23.5)	13.3 (4.4-31.7)	0.032
Disease severity				
ICU transfer, N (%)	41 (1.7%)	15 (1.1%)	26 (2.4%)	0.014
Oxygen supplementation, N (%)	28 (1.1%)	7 (0.5%)	21 (2.0%)	0.001
Pneumonia, N (%)	185 (7.5%)	73 (5.2%)	112 (10.5%)	<0.001

WBCs, white blood cells; HCT, hematocrit; MCV, mean corpuscular volume; MCH, mean corpuscular hemoglobin; MCHC, mean corpuscular hemoglobin concentration; RDW, red blood cell distribution width; PDW, platelet distribution width; CRP, c-reactive protein.

For common symptoms, hospitalized children had more often cough, sputum production, sniffle, vomiting, abdominal pain, but less fever than outpatients. Neurological (e.g., convulsion) and other symptoms (e.g., skin rash) were uncommon, but the frequencies of convulsion and skin rash in the inpatient group were noticeably higher. A substantial portion of pediatric patients were found with viral or bacterial co-infections, and those co-infections were markedly enriched among inpatients, especially for *Mycoplasma pneumoniae* and influenza A/B viruses. Most patients were previously healthy as the underlying diseases were only found few, including asthma (1.4%), allergic rhinitis (1.1%) and congenital heart defects (0.2%), predominantly among inpatients.

Hematological parameters displayed substantial abnormality and differed significantly between outpatients and inpatients. Outpatients had higher counts of WBCs, neutrophils, and monocytes, whereas lymphocyte counts were higher in inpatients. Although red blood cells or platelets related indices showed differences between outpatients and inpatients, they generally fell in the normal range. Notably, the level of CRP was deranged in most patients and became much higher among hospitalized children with significance. In addition, the inpatient group experienced significantly higher rates of adverse clinical complications than the outpatient group, including ICU transfer (2.4% vs. 1.1%, *p* = 0.014), oxygen supplementation (2.0% vs. 0.5%, *p* = 0.001), and pneumonia (10.5% vs. 5.2%, *p* < 0.001).

Multivariable logistic regression revealed that age 1-3 years and underlying comorbidity such as asthma were independently associated with increased odds of hospitalization (**Table 2**). Among presenting symptoms, sputum production, vomiting, abdominal pain, and convulsion were positively associated with hospital admission, whereas sniffle was inversely associated; fever and cough were not independently significant. Co-infection with *Mycoplasma pneumoniae* and influenza A/B virus was also associated with higher hospitalization odds, with influenza B virus infection showing the strongest effect. Of the laboratory variables, higher lymphocyte count and CRP level were independently associated with hospitalization. In subgroup analysis (**Supplementary Table 1**), patients with HAdV co-infection had a significantly higher hospitalization rate than those with mono-HAdV infection (79.8% vs. 32.0%, *p* < 0.001), as well as higher rates of pneumonia and several respiratory or systemic symptoms. However, several severity-related indices, including ICU transfer, oxygen supplementation, and CRP levels, were not significantly different between the two groups.

**Table 2:** Multivariable regression analysis of factors associated with hospitalization among pediatric patients with HAdV infection in the post-COVID group.

Factor	Odds ratio (OR)	95% Confidence interval (CI)	p
1≤Age ≤ 3	1.54	1.17-2.01	**0.002**
Asthma	38.52	4.62-321.18	**<0.001**
Symptoms			
Fever	0.91	0.59-1.41	0.679
Cough	0.79	0.61-1.03	0.081
Sputum	13.98	10.20-19.16	**<0.001**
Sniffle	0.64	0.47-0.86	**0.003**
Vomiting	2.67	1.91-3.72	**<0.001**
Abdominal pain	1.52	1.01-2.29	**0.048**
Convulsion	20.29	6.56-62.76	**<0.001**
Co-infection			
Mycoplasma pneumoniae	6.38	4.42-9.23	**<0.001**
Influenza B virus	31.81	14.39-70.34	**<0.001**
Influenza A virus	1.85	1.01-3.37	**0.046**
Peripheral blood parameters			
White blood cells	0.98	0.91-1.04	0.466
Neutrophils	0.87	0.81-0.94	**<0.001**
Lymphocytes	1.40	1.23-1.59	**<0.001**
CRP	1.02	1.02-1.03	**<0.001**

The outpatient group was used as the reference category. The odds ratios (ORs) represent the relative odds of hospitalization associated with each factor in the inpatient group compared with the outpatient group. CI, confidence interval; CRP, C-reactive protein; HAdV, human adenovirus. Bold values indicate statistically significant results (p<0.05)

In this respective study chest imaging was available in a total of 833 patients, where pulmonary abnormalities were found very common (**Supplementary Table 2**). Thickened lung texture was the predominant radiographic finding, observed in 90.2% of patients, with no significant difference between outpatients and inpatients. By contrast, findings suggestive of substantial parenchymal involvement were more frequent among inpatients, including patchy shadowing and consolidation, showing greater pulmonary involvement. Also, between outpatients and inpatients, treatment patterns varied and mirrored differences in disease burden and therapeutic intensity (**Supplementary Table 3**). Outpatient management was more often centered on symptomatic control and conventional antiviral treatment, with higher use of ibuprofen, interferon α-1b, and oseltamivir. Inpatient care, by contrast, was characterized by greater use of macrolides, penicillin, peramivir, aciclovir, and methylprednisolone, consistent with a tendency towards broader antimicrobial coverage and more intensive anti-inflammatory intervention.

### Differences in the epidemiological and clinical characteristics of hospitalized pediatric patients with HAdV infection before and after the COVID-19 pandemic

**Figure 2:**
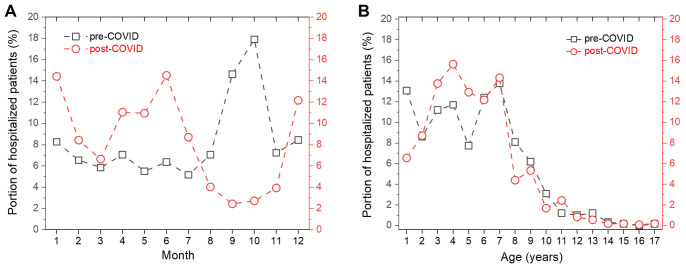
Seasonal and age-specific distributions of pediatric HAdV infection before and after the COVID-19 pandemic. **(A)** The left panel shows the monthly distribution of pediatric HAdV cases in the pre-COVID and post-COVID periods. Before the pandemic, cases were distributed relatively evenly throughout the year, with a more pronounced rise in the autumn (peaked at October). After the pandemic, the seasonality pattern shifted, with marked rises in late Spring and Winter (double peaks at June and January). **(B)** The right panel shows the age distribution of paediatric HAdV cases before and after the pandemic. Before COVID-19, cases were spread across a broader paediatric age range, whereas after COVID-19, cases became more concentrated in younger children before school age (<7 years old), with a peak in early childhood (at age of 4 years) followed by a steep decline in older age groups.

We next assessed the epidemiological changes in hospitalized pediatric patients due to the impact of the COVID-19 pandemic. A total of 581 hospitalized children were included in the pre-COVID group and 1,068 in the post-COVID group. Compared with the pre-COVID period, the epidemiological pattern of pediatric inpatients with HAdV infection substantially shifted in both seasonality and age distribution (**Figure 2**). Monthly trends were relatively stable before the pandemic, with a pronounced rise in early autumn and a peak around October, whereas after the pandemic the burden was redistributed towards winter and early summer, with higher proportions in January and from April to June, followed by a marked decline from summer into autumn (**Figure 2A**). Notably, the pre-COVID autumn surge was no longer evident in the post-COVID period.

The age profile also changed after the pandemic. Before COVID-19, hospitalizations were distributed across a broader pediatric age range, with substantial proportions extending into school-aged children (**Figure 2B**). After COVID-19, the distribution shifted towards younger children, peaking in those aged 4 years, and then declining sharply after age of 7 years (i.e., Chinese entry age for elementary school), with very few cases among older children and adolescents. These findings suggest that the post-COVID period was characterized by altered seasonal timing and concentrated HAdV infection at younger age.

For all patients included, the median age was 5 years (IQR: 3-7) with no statistical difference between the pre- and post-COVID groups (**Table 3**). Also, no differences were found in gender ratio and patient portions of those aged 1-3 years, 3 years < age <14 years, or older than 14 years between two groups. Notably, the post-COVID group had a longer hospital stay than the pre-COVID group. In terms of clinical symptoms, the post-COVID group exhibited significantly higher incidences of sputum, sore throat, vomiting, and abdominal pain, but lower frequencies of cough, convulsion, and muscle pain when compared to the pre-COVID group. In contrast, the patients in the post-COVID group experienced more co-infections with influenza A/B virus but fewer co-infections with parainfluenza virus and respiratory syncytial virus. Although most patients were previously healthy, a higher portion of children with co-existing asthma or allergic rhinitis were hospitalized in the post-COVID period.

**Table 3:** Comparison of baseline demographics, clinical characteristics and laboratory parameters between the pre-COVID and post-COVID pediatric inpatients infected with HAdV.

Characteristic	Total (n=1,649)	Pre-COVID (n=581)	Post-COVID (n=1,068)	p
Age (year)	5 (3-7)	5 (3-7)	5 (3-7)	0.882
1≤Age ≤ 3, N (%)	501 (30.4%)	191 (32.9%)	310 (29.0%)	0.105
3<Age<14, N (%)	1137 (69.0%)	386 (66.4%)	751 (70.3%)	0.116
Age≥14, N (%)	11 (0.7%)	4 (0.7%)	7 (0.7%)	0.937
Female sex, N (%)	805 (48.8%)	300 (51.6%)	505 (47.3%)	0.091
Length of stay (day)	6 (5-8)	6 (4-8)	6 (5-8)	<0.001
Symptoms				
Fever	1448 (87.8%)	503 (86.6%)	945 (88.5%)	0.258
Cough	1198 (72.7%)	461 (79.3%)	737 (69.0%)	<0.001
Sputum	752 (45.6%)	201 (34.6%)	551 (51.6%)	<0.001
Sniffle	321 (19.5%)	100 (17.2%)	221 (20.7%)	0.086
Vomiting	292 (17.7%)	84 (14.5%)	208 (19.5%)	0.011
Sore throat	143 (8.7%)	20 (3.4%)	123 (11.5%)	<0.001
Abdominal pain	122 (7.4%)	25 (4.3%)	97 (9.1%)	<0.001
Convulsion	73 (4.4%)	34 (5.9%)	39 (3.7%)	0.038
Diarrhea	57 (3.5%)	14 (2.4%)	43 (4.0%)	0.086
Muscle pain	39 (2.4%)	26 (4.5%)	13 (1.2%)	<0.001
Rash	37 (2.2%)	12 (2.1%)	25 (2.3%)	0.718
Co-infection				
Mycoplasma pneumoniae	436 (26.4%)	151 (26.0%)	285 (26.7%)	0.760
Influenza B virus	300 (18.2%)	75 (12.9%)	225 (21.2%)	<0.001
Influenza A virus	189 (11.5%)	33 (5.7%)	156 (14.6%)	<0.001
Parainfluenza virus	173 (10.5%)	156 (26.9%)	17 (1.6%)	<0.001
Respiratory syncytial virus	69 (4.2%)	37 (6.4%)	32 (3.0%)	0.001
Number of co-infections				
0	861 (52.2%)	260 (44.8%)	601 (56.3%)	<0.001
1	464 (28.1%)	192 (33.0%)	272 (25.5%)	0.001
2	235 (14.3%)	112 (19.3%)	123 (11.5%)	<0.001
3+	89 (5.4%)	17 (2.9%)	72 (6.7%)	0.001
Underlying diseases				
Asthma	43 (2.6%)	9 (1.5%)	34 (3.2%)	0.047
Allergic rhinitis	28 (1.7%)	2 (0.3%)	26 (2.4%)	0.002
Congenital heart defects	5 (0.3%)	1 (0.2%)	4 (0.4%)	0.806
Blood cell counts				
WBCs, ×10^9^/L	8.3 (5.8-11.7)	7.3 (5.5-10.3)	8.9 (6.0-12.2)	<0.001
Neutrophils, ×10^9^/L	4.7 (2.7-7.6)	3.9 (2.3-6.0)	5.2 (3.0-8.4)	<0.001
Lymphocytes, ×10^9^/L	2.3 (1.5-3.3)	2.4 (1.3-3.9)	2.2 (1.6-3.1)	0.087
Monocytes, ×10^9^/L	0.6 (0.4-0.9)	0.6 (0.4-0.8)	0.6 (0.4-1.0)	0.003
Red blood cells, ×10^12^/L	4.4 (4.2-4.7)	4.5 (4.2-4.8)	4.4 (4.2-4.6)	<0.001
Hemoglobin, g/L	121 (114-127)	122 (116-130)	120 (114-126)	<0.001
HCT, %	36.6 (34.8-38.4)	36.7 (34.9-38.5)	36.6 (34.7-38.4)	0.304
MCV, fL	83.0 (80.7-85.3)	82.1 (79.6-84.8)	83.3 (81.3-85.6)	<0.001
MCH, pg	27.3 (26.4-28.2)	27.3 (26.0-28.2)	27.3 (26.5-28.1)	0.196
MCHC, g/L	328 (320-335)	330 (322-338)	326 (319-334)	<0.001
RDW, %	12.8 (12.3-13.3)	12.9 (12.4-13.5)	12.8 (12.3-13.3)	<0.001
Platelets, ×10^9^/L	256 (206-320)	250 (191-323)	258 (213-319)	0.014
PDW, %	12.3 (10.4-15.8)	13.0 (10.7-15.8)	12.0 (10.3-15.8)	0.006
Biochemical panel				
CRP, mg/L	8.6 (2.3-24.1)	3.7 (0.8-11.5)	13.3 (4.4-31.8)	<0.001
PCT, ng/mL	0.16 (0.07-0.35)	0.12 (0.06-0.29)	0.18 (0.08-0.36)	<0.001
Total bilirubin, µmol/L	7.0 (4.8-11.2)	5.5 (3.7-8.4)	7.9 (5.5-12.3)	<0.001
Indirect bilirubin, µmol/L	3.6 (2.2-5.7)	3.3 (2.1-5.4)	3.8 (2.3-6.1)	<0.001
Direct bilirubin, µmol/L	2.7 (1.9-5.6)	1.8 (1.0-3.2)	3.6 (2.0-6.6)	<0.001
ALT, U/L	16.0 (12.8-20.0)	16.0 (12.1-22.0)	16.0 (13.0-19.6)	0.074
AST, U/L	35.0 (28.0-47.7)	39.0 (31.0-50.7)	33.0 (27.0-45.0)	<0.001
GGT, U/L	11.7 (10.0-14.0)	11.0 (9.0-14.0)	12.0 (10.0-14.4)	<0.001
Total protein, g/L	68.9 (65.5-73.5)	69.1 (66.0-73.5)	68.8 (65.1-73.5)	0.504
Albumin, g/L	42.4 (39.9-44.8)	42.6 (40.1-44.8)	42.1 (39.8-44.8)	0.240
Globulin, g/L	27.1 (23.9-30.5)	26.5 (23.8-30.0)	27.4 (23.9-30.9)	0.059
BUN, mmol/L	3.5 (2.8-4.3)	3.8 (3.0-4.6)	3.3 (2.7-4.1)	<0.001
Creatinine, μmol/L	29.8 (24.0-36.5)	27.9 (22.9-35.0)	30.6 (24.7-36.9)	<0.001
Potassium, mmol/L	4.28 (3.99-4.64)	4.34 (4.01-4.71)	4.24 (3.97-4.61)	0.010
Sodium, mmol/L	137.6 (135.2-139.5)	137.8 (135.6-139.8)	137.4 (135.0-139.4)	0.007
Calcium, mmol/L	2.30 (2.22-2.39)	2.31 (2.22-2.44)	2.29 (2.23-2.37)	<0.001
Disease severity				
ICU transfer, N (%)	37 (2.2%)	11 (1.9%)	26 (2.4%)	0.593
Oxygen supplementation, N (%)	31 (1.9%)	10 (1.7%)	21 (2.0%)	0.873
Pneumonia, N (%)	349 (21.2%)	236 (40.6%)	113 (10.6%)	<0.001

WBC, white blood cell; HCT, hematocrit; MCV, mean corpuscular volume; MCH, mean corpuscular hemoglobin; MCHC, mean corpuscular hemoglobin concentration; RDW, red blood cell distribution width; PDW, platelet distribution width; CRP, c-reactive protein; PCT, procalcitonin; ALT, alanine aminotransferase; AST, aspartate aminotransferase; GGT, γ-glutamyl transferase; BUN, blood urea nitrogen.

In the peripheral blood parameters tested, compared to the pre-COVID group, the post-COVID group had significantly higher amounts of WBCs, neutrophils and monocytes. Although most red blood cells and platelets related indices showed differences between two groups, these parameters from most patients fell into the normal range. However, for biochemical indicators, the post-COVID group demonstrated abnormally heightened levels of CRP, PCT and direct bilirubin, despite that many parameters with substantial differences between the two groups mostly fell in the normal range, such as BUN, creatinine and electrolyte (e.g., K^+^, Na^+^) concentrations. In addition, ICU transfer and oxygen supplementation were similar between the pre- and post-COVID groups, with no statistically significant differences. In contrast, pneumonia was significantly more frequent in the pre-COVID group than in the post-COVID group (40.6% vs. 10.6%, *p* < 0.001).

**Figure 3:**
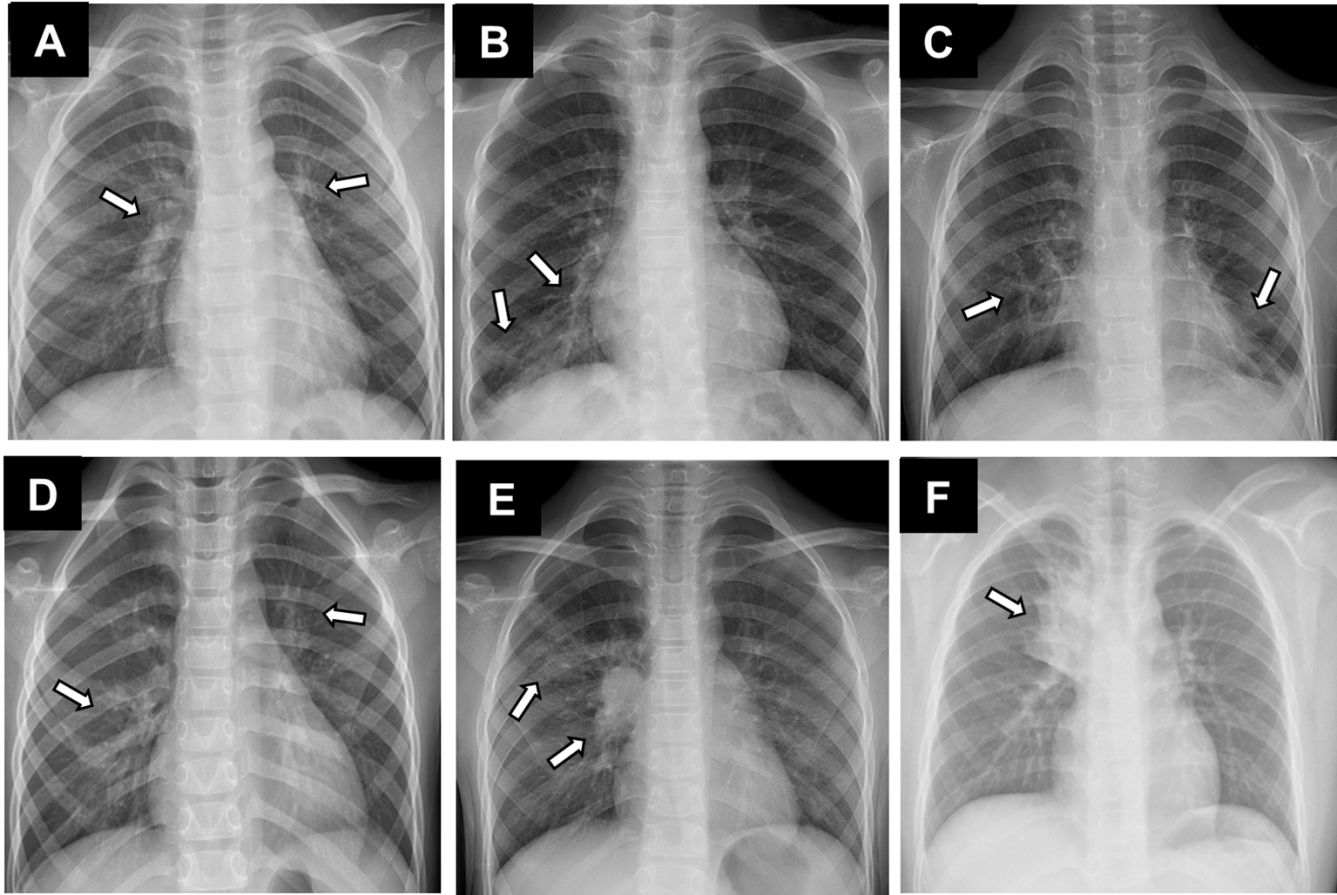
Representative X-ray graphs of pediatric inpatients with HAdV infection upon hospital admission, demonstrating typical pathological changes in the lungs from the pre-COVID group **(A–C)** to the post-COVID group **(D–F)**. **(A)** Image taken from a 5-year-old boy with fever and cough symptoms, showing increased and slightly blurred lung markings in both lungs (indicated by arrow); **(B)** Image taken from a 7-year-old boy with fever, cough, and expectoration, showing a patchy high-density shadow with poorly defined margins in the right lower lobe, and a slightly blunted right costophrenic angle (indicated by arrow); **(C)** Image taken from a 5-year-old girl having fever, cough, expectoration, and convulsion, showing increased lung markings in both lungs and patchy shadows with blurred margins (indicated by arrow); **(D)** Image taken from a 7-year-old girl with fever and cough, showing increased and blurred lung markings in both lungs (indicated by arrow); **(E)** Image taken from a 9-year-old boy with fever, cough, expectoration, and rhinorrhea, showing increased lung markings in both lungs, an enlarged and dense right hilar shadow, and a patchy shadow with blurred margins in the right upper lobe (indicated by arrow); **(F)** Image taken from a 10-year-old boy with fever, cough, and expectoration, showing a few patchy high-density shadows in the right upper lobe (indicated by arrow).

Results of multivariable regression delineated that symptoms including sputum production, sore throat, and abdominal pain, co-infection with influenza A virus, and underlying diseases exhibited significantly higher odds ratios in the post-COVID group, where cough, convulsion and red blood cell count were associated with lower odds ratios (**Table 4**). In addition, for chest X-ray imaging analysis (**Supplementary Table 4**), thickened lung texture and patchy shadow were more frequent in pre-COVID group than post-COVID group, and consolidation of lung tissue became uncommon with no significant difference between the two groups, suggesting reduced pulmonary involvement in the post-COVID patients (**Figure 3**). Furthermore, treatment patterns changed substantially from the pre-COVID to post-COVID period (**Supplementary Table 5**). Ibuprofen was used more frequently after the pandemic. Antiviral prescription also shifted, with greater use of interferon α-1b and the introduction of peramivir in the post-COVID period, whereas aciclovir use declined and oseltamivir use remained similar across the two periods. Antibiotic administration likewise changed, with cephalosporins and macrolides prescribed less often and penicillin more common after the COVID-19 pandemic. Corticosteroid use was significantly increased in the post-COVID period, including both methylprednisolone and dexamethasone.

**Table 4:** Multivariable analysis of factors differentiating the pre-COVID and post-COVID groups among hospitalized pediatric patients with HAdV infection.

Factor	OR	95% CI	p
Length of stay (day)	1.05	1.01-1.10	**0.048**
Symptoms			
Cough	0.19	0.14-0.26	**<0.001**
Sputum	5.55	4.15-7.43	**<0.001**
Sore throat	3.96	2.28-6.87	**<0.001**
Vomiting	0.77	0.54-1.08	0.133
Abdominal pain	2.24	1.31-3.83	**0.003**
Convulsion	0.53	0.31-0.92	**0.024**
Co-infection			
Influenza A virus	1.86	1.14-3.02	**0.012**
Influenza B virus	1.07	0.74-1.55	0.726
Underlying diseases			
Asthma	1.61	0.66-3.92	0.295
Allergic rhinitis	7.42	1.43-38.61	**0.017**
Blood test results			
White blood cells	0.99	0.95-1.03	0.639
Neutrophils	1.06	1.01-1.11	**0.036**
Red blood cells	0.64	0.47-0.86	**0.003**
Hemoglobin	1.00	0.99-1.00	0.410
Platelets	1.00	1.000-1.003	0.302
CRP	1.02	1.01-1.02	**<0.001**
PCT	0.99	0.85-1.15	0.875
Direct bilirubin	1.11	1.07-1.15	**<0.001**
Creatinine	1.00	0.99-1.01	0.852

The pre-COVID group was used as the reference group. Odds ratios (ORs) represent the relative odds of each factor in the post-COVID group compared with the pre-COVID group. CI, confidence interval; CRP, C-reactive protein; PCT, procalcitonin. Bold values indicate statistically significant results (p<0.05).

## Discussion

In this study we reported the epidemiological and clinical characteristics of pediatric patients with HAdV infection admitted to four tertiary hospitals in Jiangsu Province of Eastern China before and after the COVID-19 pandemic. In the post-COVID period, hospitalized patients were younger than outpatients and more frequently presented with respiratory, gastrointestinal and neurological symptoms, viral or bacterial co-infections, and raised inflammatory biomarkers, while imaging findings revealed greater pulmonary involvement compared to those in outpatients. Importantly, the epidemiological and clinical characteristics of pediatric HAdV infections have changed markedly after the COVID-19 pandemic, manifested by measurable alterations in seasonal or patient age distribution, increased influenza co-infections, and moderately heightened inflammatory responses, despite the overall clinical severity did not demonstrate a substantial deterioration.

More than 100 HAdV genotypes infect humans through respiratory, ocular, or gastrointestinal tracts (Kajon, 2024). Although mainly spreading through droplets or aerosols, HAdV transmissibility varies by genotypes from the reproductive number (R_0_) of 1.43 in one adenovirus keratoconjunctivitis outbreak caused by HAdV-54 (Omatsu et al., 2021) to a much higher R_0_ of 5.1 amid a respiratory adenovirus type 7 outbreak (Guo et al., 2020). HAdV-associated respiratory infections are concentrated in species B, C, and E, with approximately 11 well-recognized respiratory genotypes implicated in acute respiratory illness (Probst et al., 2021). To date, HAdV remains a leading cause of respiratory infection in children, accounting for approximately 4-10% of pediatric community-acquired pneumonia cases (Jain et al., 2015; Xu et al., 2023). Among respiratory adenoviruses, species B and C are most commonly implicated in pediatric disease, and genotypes HAdV-B3 and HAdV-B7 have been associated with more severe pneumonia and extrapulmonary involvement (Fu et al., 2019; Probst et al., 2025).

Mechanistically, HAdV causes illness through a combination of direct viral cytopathic injury and induced host inflammatory response (Liang et al., 2025). Infection begins with attachment of viral fiber protein to epithelial cell receptors, including the coxsackievirus-adenovirus receptor (CAR) and desmoglein-2 (DSG2), followed by internalization, intracellular trafficking, and delivery of the viral genome to the nucleus for replication (Greber and Flatt, 2019). Viral replication within epithelial cells leads to cytolysis, tissue injury, and disruption of epithelial barrier integrity; simultaneously, activation of innate immune pathways in epithelial and immune cells including NF-κB and interferon-related signaling amplifies the release of pro-inflammatory cytokines and chemokines (Liang et al., 2025; Yuan et al., 2026). In young children whose antiviral immunity is still developing, and in immunocompromised patients, this imbalance between viral replication and host defense may be more pronounced, thereby predisposing to higher viral burden, exaggerated inflammation, and more severe disease. Thus, the clinical severity of pediatric HAdV infection reflects the combined effects of host vulnerability, viral load, and dysregulated inflammatory response.

Consistent with the above explanation, our pediatric cohort in the post-COVID period showed that hospitalization was associated with younger age and higher portion of patients at age of 1-3 years. At the same time, the age distribution of HAdV infection evidently altered and became more concentrated with a distinct peak at age of 4 years, whereas before the COVID-19 pandemic cases were more broadly distributed across the preschool ages up to 7 years. This shift is consistent with the known predominance of HAdV infection in early childhood (i.e., < 5 years old) (Shen et al., 2019; Xie et al., 2019; Abu-Helalah et al., 2026) and may also reflect post-COVID changes in the susceptible population structure. The study periods (pre-COVID versus post-COVID) were defined according to key COVID-19 policy milestones in China; that is, the initiation of China’s first-level public health emergency response in January 2020, and the nationwide relaxation of COVID-19 control measures in December 2022 (Shi et al., 2023). These timepoints represent the onset of strict local interventions and the major easing of COVID-19 prevention policies in Jiangsu province, respectively. For this reason, children born during 2020-2022 in China experienced reduced social contact and changed pathogen exposure under non-pharmaceutical interventions (NPIs) against COVID-19, which may have deferred their first adenoviral exposure until restrictions were lifted at the end of 2022. Moreover, this age pattern is also compatible with resurgence of HAdV activity every 3-5 years reported in association with genotype shifts and changing transmission settings after decline during the COVID-19 pandemic (Lee et al., 2010; Shen et al., 2019; Chen et al., 2022; Kim et al., 2025).

Recently, a single-center serological study in northern China found that adenovirus antibody levels in young children were relatively stable through COVID and post-COVID periods, suggesting that the post-COVID rebound may reflect altered exposure opportunities rather than simple loss of adenovirus immunity (so-called immunity debt) (Li et al., 2026). However, the study was based on a small sample size and the research was focused on HAdV-7/-55 that were not dominant genotypes in circulation at that time in the region (Wang et al., 2023). In contrast, HAdV detection rate was significantly higher in southern China (Fang et al., 2025; Li et al., 2025), with HAdV-1/-3 dominance (Wang C. et al., 2024; Chen et al., 2025; Niu et al., 2026). Concomitantly, our study found that HAdV seasonality shifted after the COVID-19 pandemic from a predominantly unimodal autumn peak to a bimodal pattern with winter and early summer peaks. While adenovirus circulation remains regionally heterogeneous in China, recent pediatric studies similarly revealed that post-COVID activity has become less concentrated in a single season, with both summer and winter surges (Huang et al., 2025; Li et al., 2025; Zeng et al., 2025; Papini et al., 2026). This seasonality is determined by a combination of demographic, environmental and viral factors, such as geographic location and genotypic predominance. Therefore, these changes pointed out that the COVID-19 pandemic may have altered patterns of viral exposure and susceptibility, thereby reshaping the timing and age profile of HAdV-associated hospitalization.

In a 10-year retrospective study on pediatric adenovirus infections (from 2014 to 2024), the underlying neurologic disease, cardiac disease, and hematopoietic stem cell transplantation were independently associated with severe outcomes, whereas viral co-infection was common but not independently linked to worse clinical outcomes (Aykac et al., 2026). In contrast, our post-COVID analysis identified a distinct hospitalization profile characterized by more prominent multisystem symptoms (increased respiratory, gastrointestinal, and neurological manifestations), more chronic comorbidity, higher rates of *Mycoplasma pneumoniae* and influenza virus co-infection, and greater inflammatory responses as indicated by leukocytosis and elevated CRP level. Because co-infected patients showed a substantially higher hospitalization rate than those with mono-HAdV infection, hospitalization in these cases may reflect the combined effects of HAdV and co-infected pathogens. Therefore, the contribution of HAdV itself to disease severity cannot be fully separated from that of secondary or concomitant infections in this retrospective study. Nevertheless, our results are in line with previous studies identifying leukocytosis, *Mycoplasma pneumoniae* co-infection, and high blood viral load as risk factors for worsened disease severity and prolonged hospital stay (Esposito et al., 2016; Shen et al., 2019; Zhang et al., 2021; Huang et al., 2025). These differences may arise from variations in study population, outcome definition, and COVID-19 pandemic-related changes in viral circulation and host exposure (Huang et al., 2025; Zhang R. et al., 2025; Angélica Maya et al., 2026). Therefore, comorbidity should still be acknowledged as an important risk factor for severe HAdV infection, while our findings underlined that, in the post-COVID period, hospitalization profile was also re-shaped by epidemiological shifts, co-infection pattern changes, and clinical phenotype alteration rather than by comorbidity alone.

Compared with the pre-COVID period, children hospitalized with HAdV infection after the pandemic, despite similar demographic characteristics, exhibited a distinct clinical profile. They had longer hospital stays and were more likely to present with sputum production, sore throat, and abdominal pain, with a markedly higher frequency of influenza A viral co-infection and a greater prevalence of comorbidities including asthma and allergic rhinitis. The co-infection pattern is consistent with pediatric surveillance data from China showing increased influenza activity following the relaxation of NPIs (Zhang et al., 2024). Although some inflammatory markers were moderately higher in the post-COVID group, overall laboratory indices did not indicate substantial deterioration, and pulmonary manifestations revealed by chest X-ray imaging became less frequent, suggesting that the overall clinical severity of pediatric HAdV infection remained comparable between the two periods.

In immunocompetent children with HAdV infection, no routine antiviral therapy has been established, and current management remains predominantly supportive, including oxygen supplementation and mechanical ventilation, while the use of corticosteroids, intravenous immunoglobulin (IVIG), interferon-α, and oseltamivir largely depends on empirical strategies and clinician judgements rather than evidence-based HAdV-specific treatment (Soler Wenglein et al., 2025a). In addition, agents such as cidofovir are generally administered for selected severe cases or immunocompromised patients (Soler Wenglein et al., 2025a). For instance, oseltamivir is not an HAdV-directed antiviral drug and possibly reflects empirical treatment for suspected influenza-like co-infection before definitive pathogen identification. Interferon-α may have been used as an immunomodulatory antiviral strategy, but its efficacy in HAdV infection among non-immunocompromised children has not been established. Therefore, these treatments should be distinguished from HAdV-specific therapy and should not be presented as evidence-based therapeutic options for adenovirus infection.

Here the relatively high use of antibiotics and frequent chest imaging suggest that many hospitalized children were evaluated and treated empirically at an early stage when bacterial co-infection, secondary bacterial pneumonia, or lower respiratory tract involvement could not be excluded. Such an approach is consistent with real-world pediatric inpatient practice, in which antimicrobial treatment and radiographic assessment are often guided by inflammatory markers and clinical suspicion of pneumonia rather than by virological results alone. Pediatric community-acquired pneumonia guidelines also recognize the role of chest radiography in hospitalized children and support empiric antibacterial therapy when bacterial pneumonia cannot be ruled out (Bradley et al., 2011). Therefore, antibiotic use and imaging should be interpreted primarily as indicators of clinical concern, diagnostic uncertainty, and suspected bacterial co-infection or lower respiratory tract involvement, rather than as direct proxies for clinical severity. Given the observational nature of this study, the low frequency of severe outcomes (e.g., oxygen supplementation, mechanical ventilation, and ICU transfer) cannot be attributed to these interventions.

The medication patterns also changed substantially after the COVID-19 pandemic, with increased use of ibuprofen, interferon α-1b, peramivir, penicillin, and corticosteroids, but decreased use of cephalosporins, macrolides, and aciclovir. These shifts were likely multifactorial, reflecting evolving clinical experience and antimicrobial-stewardship practices, as well as changes in clinical presentation and respiratory co-infection patterns. In particular, the increased use of peramivir may correspond to the post-COVID resurgence of influenza, whereas reduced cephalosporin and macrolide use may indicate more selective antibiotic prescribing. Post-COVID alterations in the circulation and seasonality of pediatric respiratory pathogens have been widely reported and may have influenced treatment selection (Zhang et al., 2024; Zhang Y. et al., 2025). Nevertheless, because no specific antiviral therapy has been established for pediatric adenovirus infection, treatment remains largely supportive and individualized according to disease severity and suspected pathogen co-infections. Therefore, these findings should be interpreted as changes in real-world prescribing practice rather than evidence of treatment efficacy or strict adherence to specific guideline updates.

This study has several limitations. First, HAdV genotyping was not performed, which limits the abilities to explore the relationship between specific HAdV subtypes and clinical characteristics, and to infer the causal drivers of the observed post-COVID epidemiological changes. Future surveillance studies should include molecular typing to clarify whether these changes result from altered population immunity, modified transmission patterns, or shifts in the distribution of circulating HAdV genotypes. Second, although the observed post-COVID changes in seasonality and age distribution are compatible with altered exposure and susceptibility, the present study does not establish the mechanisms underlying these shifts. Third, differential testing intensity, particularly the more comprehensive testing performed among hospitalized patients and during the post-COVID period, may have increased the likelihood of detecting co-infections in these groups. Therefore, the observed co-infection rates and associated effect estimates, including ORs, may have been overestimated. Fourth, the exclusion of children younger than one year improved comparability of laboratory parameters by avoiding age-dependent differences in hematological reference intervals, but it may have underestimated the true burden of pediatric HAdV infection and limited the generalizability of our findings to the broader pediatric population. Finally, although all cases were laboratory confirmed, diagnostic methods varied over time, with antigen-based assays used throughout the study and real-time PCR for HAdV DNA introduced only in the post-COVID period. As diagnostic performance was beyond the scope of this study, potential diagnostic bias related to changing testing strategies should be considered when interpreting differences between the pre- and post-COVID periods. At the same time, testing platforms and reagents were not fully standardized across the four hospitals, which may have introduced some measurement heterogeneity. However, the reference ranges and distributions of the main laboratory results were similar among centers, suggesting that inter-hospital variability was likely limited.

## Conclusion

Pediatric HAdV infection after the COVID-19 pandemic did not simply revert to its pre-pandemic pattern, but re-emerged with a distinct epidemiological and clinical profile, marked by altered seasonality, younger age concentration, changing co-infection patterns, and distinct inpatient phenotype, while overall severity remained broadly comparable. Hospitalization was more likely in younger children and in those presenting with respiratory, gastrointestinal, or neurological manifestations, co-infection with *Mycoplasma pneumoniae* or influenza A/B viruses, and higher lymphocyte counts and CRP levels. These findings highlight the need for continued surveillance and for management strategies that remain responsive to the evolving post-COVID presentation of pediatric HAdV infection. Further studies incorporating viral genotyping, host immune profiling, and treatment outcomes are needed to refine risk stratification and optimize clinical care.

## Data Availability

The original contributions presented in the study are included in the article/**Supplementary Material**. Further inquiries can be directed to the corresponding authors.
