# Supplementary material for: Risk factors for hospitalization in pediatric patients with human adenovirus infection and changes in epidemiological and clinical characteristics before and after the COVID-19 pandemic in China: a multicenter retrospective study
Source: Front Cell Infect Microbiol. 2026 Aug 5;16:1863975. doi: 10.3389/fcimb.2026.1863975 (PMC13485729; doi:10.3389/fcimb.2026.1863975)
Supplement: Supplementary file 1 [file DataSheet1.docx]

**Supporting Information**

for

**Risk factors for hospitalization in pediatric patients with human adenovirus infection and changes in epidemiological and clinical characteristics before and after the COVID-19 pandemic in China: a multicenter retrospective study**

Xuan Xu^1^, Min Kong^2^, Xiaojun Zhang^3^, Jiangning Yin^4^, Xiu Chen^5^, Li Xie^3^, Fangling Wei^4^, Jianguo Zhang^1,*^, Zhimin Tao^1,6,*^

^1^Department of Emergency Medicine, The Affiliated Hospital, Jiangsu University, Zhenjiang, Jiangsu 212001, China.

^2^Department of Emergency Medicine, The First People’s Hospital of Kunshan, Affiliated Kunshan Hospital of Jiangsu University, Kunshan, Jiangsu 215300, China.

^3^Department of Emergency Medicine, The People’s Hospital of Danyang, Affiliated Danyang Hospital of Nantong University, Danyang, Jiangsu 212300, China.

^4^Department of Emergency Medicine, The Affiliated Jiangning Hospital of Nanjing Medical University, Nanjing, Jiangsu 211100, China.

^5^Department of Pediatrics, The People’s Hospital of Danyang, Affiliated Danyang Hospital of Nantong University, Danyang, Jiangsu 212300, China.

^6^Jiangsu Province Key Laboratory of Medical Science and Laboratory Medicine, Department of Laboratory Medicine, School of Medicine, Jiangsu University, Zhenjiang, Jiangsu 212013, China.

^*^ Correspondences should be addressed to:

Jianguo Zhang: [1000011431@ujs.edu.cn](mailto:1000011431@ujs.edu.cn)

Zhimin Tao: [jsutao@ujs.edu.cn](mailto:jsutao@ujs.edu.cn)

**Table S1. Comparison of demographic characteristics, clinical manifestations, severity indicators, and laboratory parameters between pediatric patients with HAdV mono-infection and HAdV co-infection.** Data are presented as median (interquartile range) for continuous variables or number (percentage) [*N* (%)] for categorical variables. The co-infection group included patients with HAdV and at least one additional detected pathogen, whereas the mono-HAdV group included patients with HAdV infection alone. The *p* values were calculated to compare differences between the co-infection and mono-HAdV groups.

|  | **Total (n=2,464)** | **HAdV co-infection (n=585)** | **HAdV mono-infection (n=1,879)** | ***p*** |
| --- | --- | --- | --- | --- |
| Age (year) | 5 (4-7) | 5 (3-7) | 6 (4-7) | 0.058 |
| 1≤Age≤3, *N* (%) | 549 (22.3%) | 150 (25.6%) | 399 (21.2%) | **0.029** |
| 3≤Age≤14, *N* (%) | 2178 (88.4%) | 510 (87.2%) | 1668 (88.8%) | 0.329 |
| Age≥14, *N* (%) | 18 (0.7%) | 3 (0.5%) | 15 (0.8%) | 0.667 |
| Gender, female (%) | 1179 (47.8%) | 281 (48.0%) | 898 (47.8%) | 0.814 |
| **Symptoms** |  |  |  |  |
| Fever | 2282 (92.6%) | 536 (91.6%) | 1746 (92.9%) | 0.338 |
| Cough | 1352 (54.9%) | 414 (70.8%) | 938 (49.9%) | **<0.001** |
| Sputum | 677 (27.5%) | 276 (47.2%) | 401 (21.3%) | **<0.001** |
| Sniffle | 464 (18.8%) | 142 (24.3%) | 322 (17.1%) | **<0.001** |
| Vomiting | 352 (14.3%) | 116 (19.8%) | 236 (12.6%) | **<0.001** |
| Sore throat | 309 (12.5%) | 84 (14.4%) | 225 (12.0%) | 0.147 |
| Stomachache | 184 (7.5%) | 54 (9.2%) | 130 (6.9%) | 0.077 |
| Diarrhea | 94 (3.8%) | 18 (3.1%) | 76 (4.0%) | 0.345 |
| Muscle pain | 75 (3.0%) | 16 (2.7%) | 59 (3.1%) | 0.719 |
| Convulsion | 44 (1.8%) | 21 (3.6%) | 23 (1.2%) | **<0.001** |
| Rash | 33 (1.3%) | 13 (2.2%) | 20 (1.1%) | 0.055 |
| **Underlying diseases** |  |  |  |  |
| Asthma | 35 (1.4%) | 16 (2.7%) | 19 (1.0%) | **0.004** |
| Allergic rhinitis | 26 (1.1%) | 18 (3.1%) | 8 (0.4%) | **<0.001** |
| **Outcomes** |  |  |  |  |
| Oxygen supplement, *N* (%) | 28 (1.1%) | 11 (1.9%) | 17 (0.9%) | 0.085 |
| Pneumonia, *N* (%) | 185 (7.5%) | 97 (16.6%) | 88 (4.7%) | **<0.001** |
| Hospitalization, *N* (%) | 1068 (43.3%) | 467 (79.8%) | 601 (32.0%) | **<0.001** |
| ICU transfer, *N* (%) | 41 (1.7%) | 12 (2.1%) | 29 (1.5%) | 0.513 |
| **Peripheral blood parameters** |  |  |  |  |
| WBCs, ×10^9^/L | 10 (7.2-12.9) | 8.9 (6.5-12.1) | 10.3 (7.7-13.0) | **<0.001** |
| Neutrophils, ×10^9^/L | 6.7 (4.3-9.5) | 5.6 (3.5-8.6) | 6.9 (4.6-9.6) | **<0.001** |
| Lymphocytes, ×10^9^/L | 2.0 (1.5-2.7) | 2 (1.5-2.8) | 2.0 (1.5-2.6) | 0.865 |
| Monocytes, ×10^9^/L | 0.8 (0.6-1.1) | 0.7 (0.5-1.0) | 0.8 (0.6-1.1) | **<0.001** |
| Red blood cells, ×10^12^/L | 4.5 (4.3-4.8) | 4.4 (4.2-4.7) | 4.6 (4.3-4.8) | **<0.001** |
| Hemoglobin, g/L | 124 (118-131) | 123 (116-127) | 125 (118-131) | **<0.001** |
| HCT, % | 38.9 (36.5-41.2) | 37.4 (35.1-39.6) | 39.4 (37.1-41.5) | **<0.001** |
| MCV, fL | 85.7 (82.8-88.5) | 84.1 (81.5-86.9) | 86.1 (83.3-88.8) | **<0.001** |
| MCH, pg | 27.4 (26.6-28.2) | 27.5 (26.7-28.3) | 27.4 (26.6-28.2) | **0.047** |
| MCHC, g/L | 318 (312-326) | 326 (318-334) | 317 (311-324) | **<0.001** |
| RDW, % | 12.9 (12.5-13.4) | 12.9 (12.4-13.4) | 12.9 (12.5-13.4) | 0.723 |
| Platelets, ×10^9^/L | 254 (213-305) | 259 (215-321) | 251 (211-300) | **<0.001** |
| PDW, % | 12.6 (10.4-15.9) | 15.3 (10.5-15.9) | 12.3 (10.3-15.8) | **0.013** |
| CRP, mg/L | 12.7 (4.5-26.4) | 12.8 (4.3-31.2) | 12.7 (4.6-25.4) | 0.261 |

**Abbreviations:** HAdV, human adenovirus; WBCs, white blood cells; HCT, hematocrit; MCV, mean corpuscular volume; MCH, mean corpuscular hemoglobin; MCHC, mean corpuscular hemoglobin concentration; RDW, red blood cell distribution width; PDW, platelet distribution width; CRP, C-reactive protein.

**Table S2.** Chest X-ray findings in a part of post-COVID HAdV-infected pediatric patients.

|  | **Total (n=833)** | **Outpatient (n=439)** | **Inpatient (n=394)** | ***p*** |
| --- | --- | --- | --- | --- |
| **Imaging manifestations** |  |  |  |  |
| Thickened lung texture | 751 (90.2%) | 403 (91.8%) | 348 (88.3%) | 0.093 |
| Patchy shadow | 182 (21.8%) | 73 (16.6%) | 109 (27.7%) | **<0.001** |
| Consolidation | 13 (1.6%) | 1 (0.2%) | 12 (3.0%) | **0.001** |

**Table S3.** Medications of pediatric HAdV-infected patients in the post-COVID period.

|  | **Total (n=2,464)** | **Outpatient (n=1,396)** | **Inpatient (n=1,068)** | ***p*** |
| --- | --- | --- | --- | --- |
| **Ibuprofen** | 908 (36.9%) | 560 (40.1%) | 348 (36.7%) | **<0.001** |
| **Antivirals** |  |  |  |  |
| Interferon α-1b | 1149 (46.6%) | 757 (54.2%) | 392 (36.7%) | **<0.001** |
| Oseltamivir | 364 (14.7%) | 312 (22.4%) | 52(4.9%) | **<0.001** |
| Peramivir | 209 (8.5%) | 19 (1.4%) | 190 (17.8%) | **<0.001** |
| Aciclovir | 18 (0.7%) | 3 (0.2%) | 15 (1.4%) | **<0.001** |
| **Antibiotics** |  |  |  |  |
| Cephalosporin | 1189 (48.3%) | 707 (50.6%) | 482 (45.1%) | **0.007** |
| Macrolides | 815 (33.1%) | 324 (23.2%) | 491 (46.0%) | **<0.001** |
| Penicillin | 736 (29.9%) | 349 (25.0%) | 387 (36.2%) | **<0.001** |
| **Corticosteroid** |  |  |  |  |
| Methylprednisolone | 419 (17.0%) | 110 (7.9%) | 309 (28.9%) | **<0.001** |
| Dexamethasone | 360 (14.6%) | 224 (16.1%) | 136 (12.7%) | **0.021** |

**Table S4.** Chest X-ray findings of pediatric inpatients infected with HAdV in the pre-COVID vs. post-COVID groups.

|  | **Total (n=802)** | **Pre-COVID (n=408)** | **Post-COVID (n=394)** | ***p*** |
| --- | --- | --- | --- | --- |
| **Imaging manifestations** |  |  |  |  |
| Thickened lung texture | 731 (91.1%) | 383 (93.9%) | 348 (88.3%) | **0.006** |
| Patchy shadow | 345 (43.0%) | 236 (57.8%) | 109 (27.7%) | **<0.001** |
| Consolidation | 17 (2.1%) | 5 (1.2%) | 12 (3.0%) | 0.074 |

**Table S5.** Medications of pediatric inpatients infected with HAdV in the pre-COVID vs. post-COVID groups.

|  | **Total (n=1,649)** | **Pre-COVID (n=581)** | **Post-COVID (n=1,068)** | ***p*** |
| --- | --- | --- | --- | --- |
| **Ibuprofen** | 480 (29.1%) | 132 (22.7%) | 348 (32.6%) | **<0.001** |
| **Antivirals** |  |  |  |  |
| Interferon α-1b | 460 (27.9%) | 68 (11.7%) | 392 (36.7%) | **<0.001** |
| Peramivir | 190 (11.5%) | 0 | 190 (17.8%) | **<0.001** |
| Oseltamivir | 74 (4.5%) | 22 (3.8%) | 52 (4.9%) | 0.416 |
| Aciclovir | 45 (2.7%) | 30 (5.2%) | 15 (1.4%) | **<0.001** |
| **Antibiotics** |  |  |  |  |
| Cephalosporin | 948 (57.5%) | 464 (79.9%) | 482 (45.1%) | **<0.001** |
| Macrolides | 946 (57.4%) | 457 (78.7%) | 491 (46.0%) | **<0.001** |
| Penicillin | 460 (27.9%) | 73 (12.6%) | 387 (36.2%) | **<0.001** |
| **Corticosteroid** |  |  |  |  |
| Methylprednisolone | 435 (26.4%) | 126 (21.7%) | 309 (28.9%) | **<0.001** |
| Dexamethasone | 152 (9.2%) | 16 (2.8%) | 136 (12.7%) | **<0.001** |
